# Post-activation potentiation effect of eccentric overload and traditional weightlifting exercise on jumping and sprinting performance in male athletes

**DOI:** 10.1371/journal.pone.0222466

**Published:** 2019-09-12

**Authors:** Marco Beato, Alexander E. J. Bigby, Kevin L. De Keijzer, Fabio Y. Nakamura, Giuseppe Coratella, Stuart A. McErlain-Naylor

**Affiliations:** 1 School of Health and Sports Sciences, University of Suffolk, Ipswich, United Kingdom; 2 Associate Graduate Program in Physical Education UPE/UFPB, João Pessoa, PB, Brazil; 3 The College of Healthcare Sciences, James Cook University, Townsville, Australia; 4 Department of Biomedical Sciences for Health, University of Milan, Milan, Italy; Instituto Politecnico de Viana do Castelo, PORTUGAL

## Abstract

The aim of this study was to evaluate the post-activation potentiation (PAP) effects following eccentric overload (EOL) and traditional weightlifting (TW) exercise on standing long jump (SLJ), countermovement jump (CMJ), and 5 m sprint acceleration performance. Ten male athletes were involved in a randomized, crossover study. The subjects performed 3 sets of 6 repetitions of EOL or TW half squat exercise followed by SLJ, CMJ, and 5 m sprint tests at 1 min, 3 min and 7 min, in separate sessions using a randomized order. Bayes factor (BF_10_) was reported to show the strength of the evidence. Differences were found using EOL for SLJ distance at 3 min (BF_10_ = 7.24, +8%), and 7 min (BF_10_ = 19.5, +7%), for CMJ at 3 min (BF_10_ = 3.25, +9%), and 7 min (BF_10_ = 4.12, +10.5%). Differences were found using TW exercise for SLJ at 3 min (BF_10_ = 3.88, +9%), and 7 min (BF_10_ = 12.4, +9%), CMJ at 3 min (BF_10_ = 7.42, +9.5%), and 7 min (BF_10_ = 12.4, *+*12%). No meaningful differences were found between EOL and TW exercises for SLJ (BF_10_ = 0.33), CMJ (BF_10_ = 0.27), and 5 m sprint (BF_10_ = 0.22). In conclusion, EOL and TW exercises acutely increase SLJ and CMJ, but not 5 m sprint performance. The PAP time window was found between 3 min and 7 min using both protocols. This study did not find differences between EOL and TW exercises, and so both methodologies can be used to stimulate a PAP response.

## Introduction

Post-activation potentiation (PAP) is a physiological phenomenon associated with an acute improvement in muscular performance after a resistance training protocol [1,2]. Neuromuscular, mechanical and biochemical changes may induce these temporary improvements in performance but the exact underlying mechanisms are still not fully understood [1,3]. The most strongly supported explanation for the effects of PAP relates to a greater rate of cross-bridge attachment as a result of phosphorylation of myosin regulatory light chains during muscle contraction [4]. Furthermore, PAP is proposed to result from increased sensitivity of contractile proteins to calcium (Ca^2+^) released from the sarcoplasmic reticulum, resulting in a cascade of events leading to an enhanced muscular response [3–5].

PAP may be induced through the use of resistance training exercises prior to the main sport-specific activity, leading to an increase in performance [6]. Generally, following a pre-load exercise, a temporary fatigue-induced decrement in performance is observed, which is subsequently replaced by a PAP response [3,7]. Traditional weightlifting (TW) is one of the modalities used by coaches to elicit a PAP response for subsequent competitive activities [3]. The majority of research investigating TW and its PAP response has reported a positive effect on reducing short distance sprint time and improving countermovement jump (CMJ) performance [3,8]. Both heavy and moderate load back squat (90% and 60% 1RM, respectively) have been shown to potentiate the sprinting and jumping performance of male professional rugby players [9]. Some previous studies on acute lower limb performance have found positive improvements after traditional pre-load strategies, while others have failed to confirm such results [3]. Such discrepancies may be a result of differences in the interventions relating to protocol characteristics including exercise modality, volume, intensity, muscle action, and duration of rest between the pre-load exercise and the subsequent sport-specific task, all of which have been identified as key variables determining the magnitude of a PAP response [6,10].

Flywheel ergometers are commonly used in sports training to chronically improve elite soccer players’ jump and sprint performances [1,11,12]. Such devices are capable of stimulating an eccentric overload (EOL), in which the generated eccentric muscular force exceeds the maximal concentric force [13,14]. The user rotationally accelerates the flywheel with maximal velocity during the concentric phase of the movement (*e*.*g*. extension phase of a squat), resulting in a flywheel inertial torque that imparts high linear resistance during the subsequent eccentric phase of the movement (*e*.*g*. flexion phase of a squat) [1,11,12]. The main advantage of this exercise methodology is related to the high mechanical overload of the eccentric phase, which may enable strength and conditioning practitioners to improve athletes’ performances both chronically and acutely [7,12]. Indeed, the greater eccentric load may recruit higher order motor units or fast-twitch muscle fibers at a greater extent and therefore likely facilitate a greater PAP response in subsequent sport-specific performance [4]. Moreover, eccentric load generated by a flywheel device may contribute to acutely improving stretch-shortening cycle performance and transfer effects on the explosive athletic tasks such as vertical jumps, horizontal jumps and sprinting [7,14,15].

Very few studies have evaluated the acute PAP induced improvement in lower limb performance following flywheel exercise [16]. Recently, acute sprint (20 m) and CMJ performance improvements have been found after EOL exercise [1]. Similar EOL-induced PAP improvements were reported in quadriceps concentric peak torque, hamstring concentric and eccentric peak torque during an isokinetic test (60°^.^s^-1^) [7]. Moreover, augmented CMJ height, impulse, peak power and peak force were observed following the same EOL exercise protocol [7]. This study reported that PAP improves lower limb performance after 3 minutes of recovery following a flywheel squat exercise, with optimal time windows from 3 to 9 min. Previous studies using TW exercises have revealed inconsistent findings since several confounding factors may affect PAP response [4]. Indeed, PAP response may be affected by subjects’ resistance training experience and competitive level [3,8]. It is not currently well established whether EOL is a more beneficial methodology to increase PAP and consequent lower-limb performance than TW, or *vice versa*. Such a comparison may have several practical applications in strength and conditioning in sport as well as for warm-up strategies before some competitions.

EOL and TW may be valid strategies to elicit acute PAP mediated improvements in lower limb power and therefore may play a functional role in sports performance training. Standing long jump (SLJ), CMJ and sprinting are well established tests to assess the lower-limb capacities. The aims of this study were: firstly, to study the acute effect of EOL and TW exercise on such sport-specific tasks; and secondly, to compare the magnitude of such acute effects between EOL and TW exercises. Such knowledge may be relevant for practitioners in order to generate PAP strategies prior to competition and training. Considering the greater peak power generated during the eccentric phase of the squat exercise, it could be supposed that EOL exercise may produce a higher PAP response in the subsequent sport-specific tasks than TW. However, authors hypothesize that both protocols should stimulate a positive PAP response in jumping and sprinting performance.

## Materials and methods

### Subjects

Ten male amateur athletes were enrolled in this study (mean ± SD: age 22 ± 2 years; body mass 73.2 ± 8.0 kg; height 1.79 ± 0.05 m) with ≥ 4 years experience with heavyweight training at a regional level. Inclusive criteria for participation were the absence of any injury or illness and regular participation in training activities (a minimum of 3 training sessions per week) as used in previous research [7]. A Bayesian adaptive sample size approach was used in this study to estimate the number of subjects [17] based on previous research of the same group [14]. Subjects were familiar with TW and EOL exercises and test procedures. All subjects were informed about the potential risks and benefits of the current procedures and gave their written informed consent. The Ethics Committee of the School of Science, Technology, and Engineering, University of Suffolk (UK) approved this study. All procedures were conducted according to the Declaration of Helsinki for studies involving human subjects.

### Experimental overview

**Fig 1:**
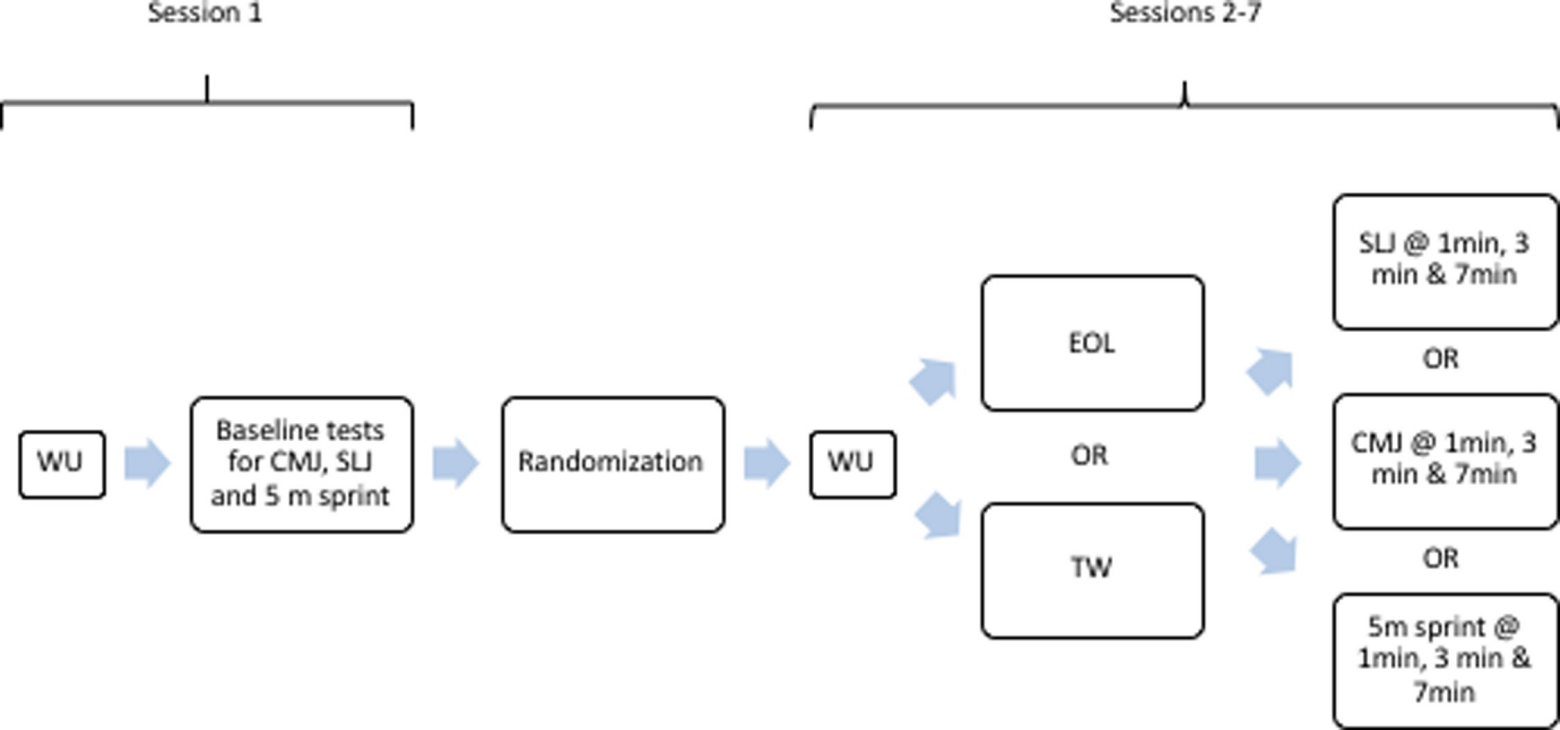
Experimental procedure. CMJ = Countermovement jump, SLJ = Standing Long jump, min = minutes, EOL = Eccentric overload, TW = Traditional weightlifting.

The acute effects of EOL *vs* TW exercise on SLJ, CMJ, and 5 m sprint performance were investigated in the present randomized, cross-over study design. Each subject attended the laboratory on seven separate occasions. This was necessary to remove a possible transient fatigue effect. The sessions were separated by 48 h of recovery to allow an adequate recovery period. Researchers required subjects to maintain their normal nutritional intake during the experimental period. Alcohol and caffeine were not permitted prior to the experimental sessions but hydration was allowed during the sessions. In the first session subjects performed the baseline condition and familiarization to EOL and TW [14]. During the baseline conditions athletes performed the same warm-up protocol utilized during the experimental condition but without any pre-load exercise (neither EOL or TW). In each of the following sessions (sessions were performed in a randomized order using Randomization.com, in order to remove any possible learning effect), the subjects performed the warm-up procedure utilized during the baseline condition followed by one of the two exercise modalities (EOL and TW). At 1 min, 3 min and 7 min after completion of the final EOL or TW set, one of the three performance tests (SLJ, CMJ, or 5 m linear sprint acceleration) were performed to evaluate the PAP effect (procedure reported in Fig 1). The authors considered that use of this protocol limited the confounding effect of repeated jumps as previously reported [7,14]. These time windows were used to observe PAP optimization, as used with success in previous studies [3,7].

### Procedures

Body mass and height were recorded by Stadiometer (Seca 286dp, Hamberg, Germany). A standardized warm-up was conducted each session, including 10 min of cycling at a constant power (1 W per kg of subject’s body mass) on an ergometer (Sport Excalibur lode, Groningen, Netherland). Dynamic mobilization was performed in both the baseline and experimental conditions. Mobilization was performed immediately after the cycling warm-up for a duration of 3 minutes and consisted of dynamic movements mimicking the exercise (*e*.*g*. half squat) and dynamic hip, knee, and ankle movements. Such procedure was utilized prior to baseline tests as previously utilized by the same research group [14].

A SLJ was utilized to test the explosive horizontal power capabilities of the lower limb musculature, as previously reported [18]. Subjects performed one maximal bilateral anterior jump with arm swing. Jump distance was measured from the starting line to the point at which the heel contacted the ground on landing [19]. The validity of this test was previously reported in the literature involving a sample of physical education students [20]. An *excellent* (ICC = 0.90) baseline test-retest intrasession reliability was found in the current study. The smallest worthwhile change (SWC) was 5 cm for SLJ.

CMJ height was investigated using an infrared device (OptoJump, Microgate, Bolzano, Italy). The subjects were instructed to stand, lower themselves to a self-selected depth and immediately jump. Arms were placed on the hips to minimize the confounding effects of arm swing and the subjects were instructed to minimize knee flexion before landing. An *excellent* (ICC = 0.92) baseline test-retest intrasession reliability was found in the current study. The SWC was 1.2 cm for CMJ.

Five-meter sprints were performed to evaluate improvements in acceleration ability. Infrared timing gates (Microgate, Bolzano, Italy) were placed at the start and end of a measured 5 m distance. On the “Go” command, the subjects were instructed to sprint through the timing gates positioned as previously reported in literature [21]. No countermovement before the sprint was permitted. A *good* (ICC = 0.86) test-retest intrasession reliability was found in the current study. The SWC was 0.03 s for 5 m sprint performance.

### Intervention

EOL half squat exercise was performed using a flywheel ergometer (D11 Full, Desmotec, Biella, Italy). The protocol consisted of 3 sets x 6 repetitions of half squats, interspersed by 2 min of passive recovery [7]. The subjects were instructed to perform the concentric phase with maximal velocity and to control the eccentric phase until the knees where flexed to approximately 90° [14]. The following load was used for each subject: one Pro disc (diameter = 0.285 m; mass = 6.0 kg; moment of inertia = 0.06 kg^.^m^2^) based on previous published research [14]. The moment of inertia of the ergometer was estimated as 0.0011 kg^.^m^2^. Power was calculated for each repetition using an integrated rotary position transducer.

TW was performed as a half squat exercise using an Olympic bar. The PAP protocol consisted of 3 sets x 6 repetitions of half squats, interspersed by 2 min of passive recovery [3]. The subjects were instructed to perform the concentric phase with maximal velocity and to control the eccentric phase until the knees were flexed to approximately 90° [22]. During the familiarization session, the TW squat loads were adjusted in order to match the peak concentric power production between TW and EOL. This was achieved by increasing the barbell load by 5 kg until the concentric peak power was within 10% of that of the EOL. The mean load was 57.7 ± 10.1 kg. Lower limb power was assessed during TW exercise by a linear position transducer (Cronojump, Barcelona, Spain).

The EOL (1097 ± 341 W, 14.98W/Kg) and TW (1030 ± 298 W, 14.07 W/Kg) concentric peak power during load matching were not meaningfully different between the conditions: Bayes factor (BF_10_) = 0.88 (*anecdotal*; effect size = 0.51; 95% credible interval [CI]: -0.20, 1.35). The EOL (1138 ± 263 W) and TW (798 ± 286 W) eccentric power were meaningfully different: BF_10_ = 44.42 (*very strong*; effect size = 1.88; 95% CI: 0.61, 3.05).

### Statistical analysis

Data were presented as mean ± SD. The test–retest intrasession reliability (during baseline session) was assessed using an intraclass correlation coefficient (ICC) and interpreted as follows: ICC ≥ 0.9 = *excellent*; 0.9 > ICC ≥ 0.8 = *good*; 0.8 > ICC ≥ 0.7 = *acceptable*; 0.7 > ICC ≥ 0.6 = *questionable*; 0.6 > ICC ≥ 0.5 = *poor*; ICC < 0.5 = *unacceptable* [23]. A fully Bayesian statistical approach to provide probabilistic statements was used in this study [24]. Each analysis was conducted with a “noninformative” prior (Cauchy, 0.707). Bayesian repeated measures ANOVA was used to evaluate the effects of time (within; baseline, 1 min, 3 min, 7 min) and exercise modality (between; EOL vs TW) on each of SLJ, CMJ, and 5 m sprint performance. If a meaningful BF_10_ was found, a Bayesian post-hoc was performed [25]. Markov Chain Monte Carlo with Gibbs sampling was used to make inferences (10000 samples) [26]. Estimates of median standardized effect size and 95% credible interval were calculated. Evidence for the alternative hypothesis (H_1_) was set as BF_10_ > 3 and evidence for null hypothesis was set as BF_10_ < 1/3 [27]. BF_10_ was reported to indicate the strength of the evidence for each analysis (between and within). The BF_10_ was interpreted using the following evidence categories: 1 < BF_10_ < 3 = *anecdotal* evidence for H_1_; BF_10_ ≥ 3 = *moderate*; BF_10_ ≥ 10 = *strong*; BF_10_ ≥ 30 = *very strong*; BF_10_ ≥ 100 = *extreme* [27]. SWC was calculated as 0.2 x SD for SLJ, CMJ and 5 m sprint performance. Statistical analyses were performed within JASP (Amsterdam, Netherland) software Version 0.9.1.

## Results

**Fig 2:**
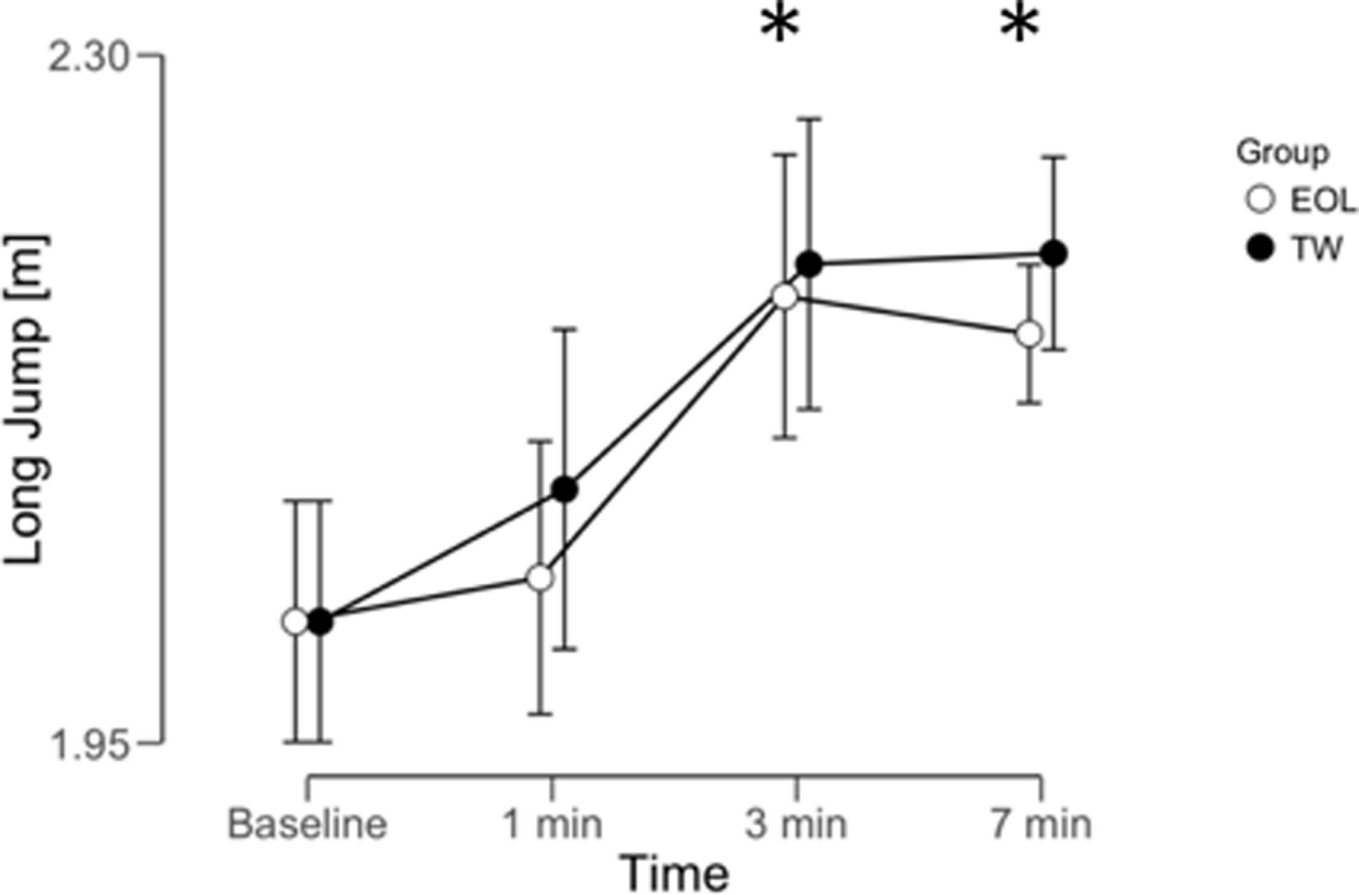
PAP time window on long jump performance following eccentric overload (EOL) and traditional weightlifting (TW) exercise. Data reported as mean ± 95% credible interval (n = 10). * = meaningful difference compared to baseline for both protocols.

**Fig 3:**
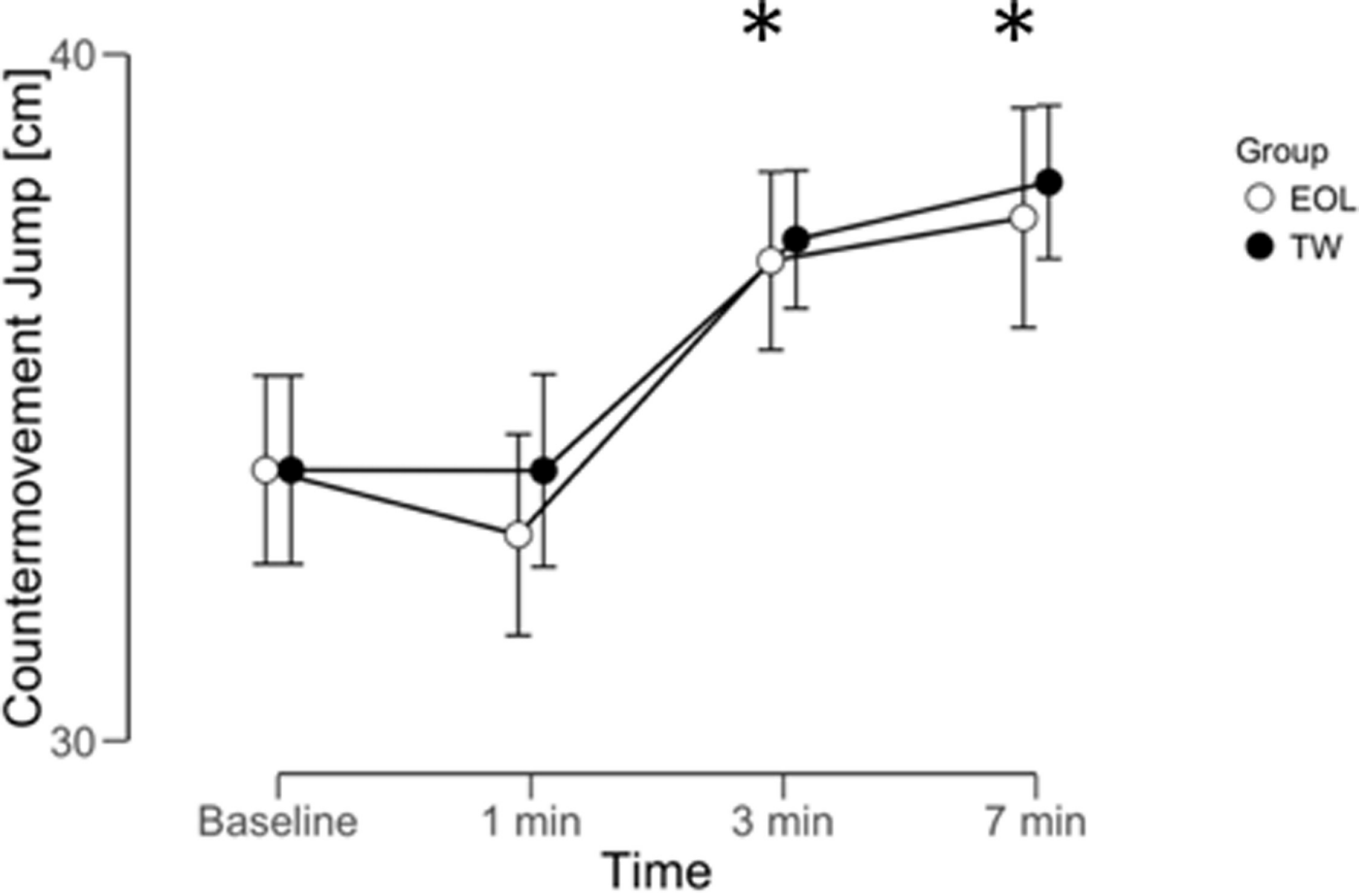
PAP time window on countermovement jump performance following eccentric overload (EOL) and traditional weightlifting (TW) exercise. Data reported as mean ± 95% credible interval (n = 10). * = meaningful difference compared to baseline for both protocols.

**Fig 4:**
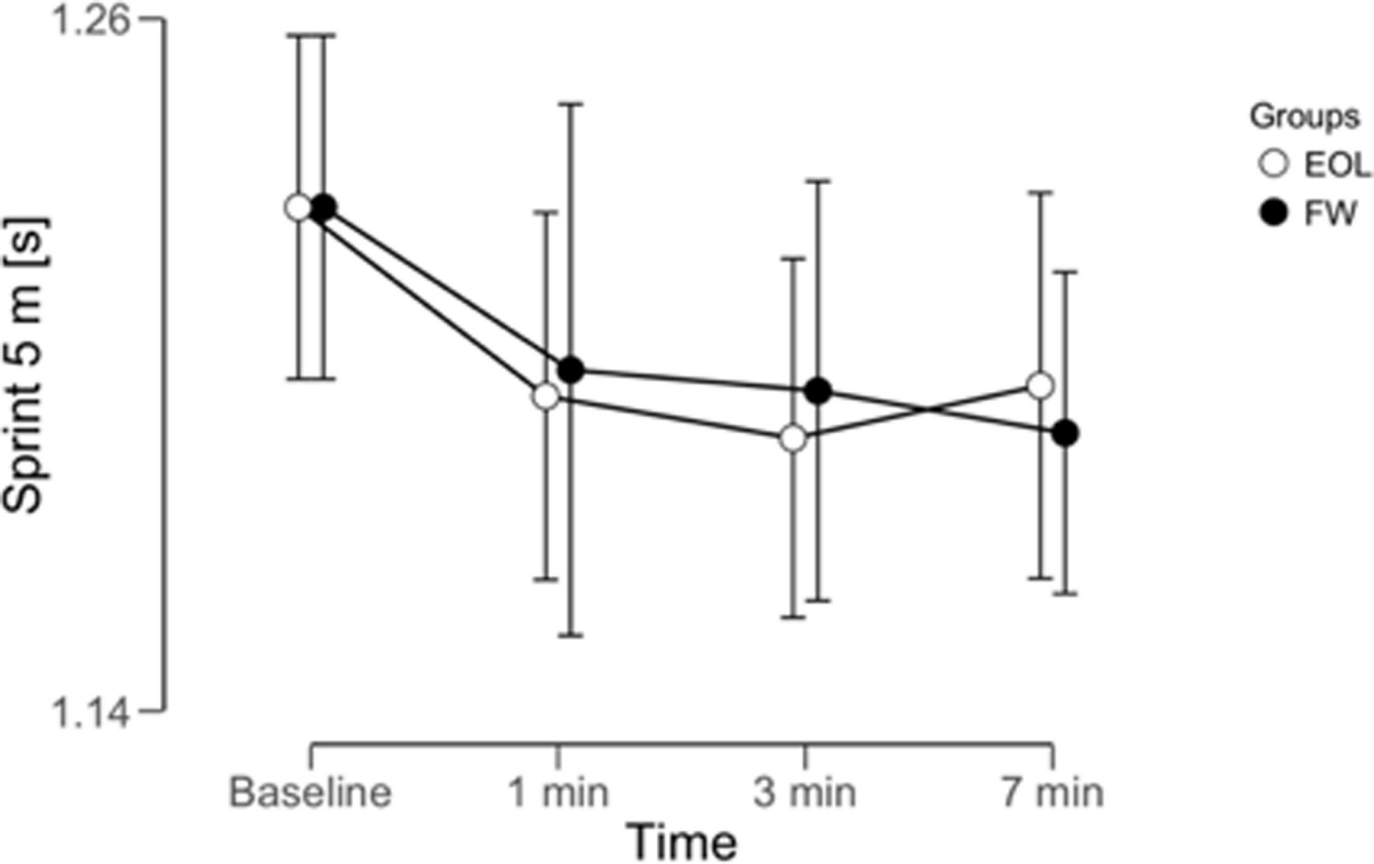
PAP time window on sprint 5 m performance following eccentric overload (EOL) and traditional weightlifting (TW) exercise. Data reported as mean ± 95% credible interval (n = 10). * = meaningful difference compared to baseline for both protocols.

The repeated ANOVA reported differences within (time) using EOL exercise for SLJ (BF_10_ = 354.2, *extreme*), CMJ height (BF_10_ = 698.3, *extreme*), but not in 5 m sprint (BF_10_ = 0.61, *anecdotal*). The repeated ANOVA reported differences within (time) using TW exercise for SLJ (BF_10_ = 193.1, *extreme*), CMJ height (BF_10_ = 6967.3, *extreme*), but not in 5 m sprint (BF_10_ = 0.37, *anecdotal*). A graphical representation of time effect on SLJ, CMJ and 5 m sprint was reported in Figs 2–4. No meaningful time x condition interactions were reported for any parameter analyzed: SLJ (BF_10_ = 0.182, *moderate* in favor of H_0_); CMJ (BF_10_ = 0.159, *moderate* in favor of H_0_); 5 m sprint (BF_10_ = 0.049, *moderate* in favor of H_0_). The repeated ANOVA did not report meaningful differences between (conditions) EOL and TW exercise for SLJ (BF_10_ = 0.33, *moderate* in favor of H_0_), CMJ (BF_10_ = 0.27, *moderate* in favor of H_0_), or 5 m sprint (BF_10_ = 0.218, *moderate* in favor of H_0_).

Bayesian post-hoc analysis comparing baseline values and time following EOL was reported for the following parameters: SLJ at 1 min (BF_10_ = 0.165, *moderate* in favor of H_0_), 3 min (BF_10_ = 7.24, *moderate*, +8%), and 7 min (BF_10_ = 19.5, *strong*, +7%); CMJ at 1 min (BF_10_ = 0.19, *moderate* in favor of H_0_), 3 min (BF_10_ = 3.25, *moderate*, +9%), and 7 min (BF_10_ = 4.12, *moderate*, +10.5%). Bayesian post-hoc analysis comparing baseline values and time following TW was reported for the following parameters: SLJ at 1 min (BF_10_ = 0.22, *moderate* in favor of H_0_), 3 min (BF_10_ = 3.88, *moderate*, +9%), and 7 min (BF_10_ = 12.4, *moderate*, +9%); CMJ at 1 min (BF_10_ = 0.12, *moderate* in favor of H_0_), 3 min (BF_10_ = 7.42, *moderate*, +9.5%), and 7 min (BF_10_ = 12.4, *strong*, +12%). Post-hoc analysis regarding 5 m sprint was not performed since no time effect was reported.

## Discussion

To the best of the authors’ knowledge, this study is the first to investigate the PAP response following EOL and TW exercises on SLJ, CMJ and 5 m sprint tasks. This study compares, also for the first time, PAP magnitude between EOL and TW exercises on functional lower limb tests, matching concentric peak power between the exercises. The present study showed that a meaningful positive PAP response can be observed after 3 min of recovery (and persists until at least 7 min) following both EOL and TW exercises on SLJ and CMJ performance but not on 5 m sprint performance in male amateur athletes. Furthermore, meaningful evidence (in favor of H_0_) revealed no differences in PAP response for each performance variable analyzed between EOL and TW protocols, therefore both protocols exhibited similar PAP responses. These findings may have an important impact on practitioners’ strength training strategies in order to develop PAP and enhance its magnitude and time window.

PAP is a physiological phenomenon that can be observed following a pre-load strategy and has previously been identified as a strength–power–potentiation complex [8]. Previous studies reported that PAP effects on CMJ performance (*e*.*g*. jump height, peak power, and impulse) and short sprinting tasks may be obtained after an EOL exercise [1,7]. Similarly, the literature supports such positive improvements (*e*.*g*. horizontal and vertical jump performance) following TW exercises [28]. For example, TW back squat, using different intensities (*e*.*g*. moderate or heavy), may induce a positive PAP response on jumping activities following a recovery period [3]. The current study supports the general knowledge that PAP is observed following a recovery period [4]. Therefore, a passive recovery of around 3 min seems to be sufficient between the pre-load strategy (*e*.*g*. EOL or TW) and the following sport-specific task (*e*.*g*. SLJ, CMJ) in order to observe performance benefits. Previous evidence reported that PAP time windows may be altered using different pre-load strategies [8]. However, the current study finds similar PAP time windows between EOL and TW exercises [4,6,7]. Future investigations, however, are needed to better clarify the optimal onset of PAP (*e*.*g*. lower volume protocols may be beneficial for PAP without stimulating as much acute fatigue) since the current study used 3 sets of 6 repetitions. This study reports that following an acute improvement in sport-specific tasks at 3 min, such positive effects were confirmed at 7 min using both EOL and TW exercises. Such a result is in line with the major body of evidence in the literature reporting an optimal time window between 3 and 10 min [7,29,30]. A recent meta-analysis found that the recovery time affects PAP magnitude, and that the optimal time window should be between 4 to 8 minutes, while a shorter recovery time (*e*.*g*. 1 min) may reduce the PAP effect on sport specific tasks [8].

The findings reported in the current study support the previous knowledge on performance improvements following a pre-load exercise except for 5 m sprint, for which a PAP effect was not found following either EOL or TW exercise. This result may be attributable to the following observations: 5 m may not be a suitable sprint distance to assess PAP and a longer (*e*.*g*. 10 or 20 m) sprinting distance could be more suitable (reliability of the sprint test increases with distance) [21]; from a biomechanical perspective short accelerations may be affected by subjects’ coordination, which could have impaired the 5 m sprint PAP response obtained with the present protocols; and finally, because both EOL and TW exercises were not biomechanically similar to the sprint, which may have limited the transfer to sprinting capacity. Indeed, the kinetic responses to a pre-load exercise may be related to its specific directional loading nature (*e*.*g*. vertical loading during a squat exercise) [31]. Therefore, a different exercise such as a barbell hip trust or a single step acceleration using a flywheel may be more effective for acute sprinting improvements due to the more horizontal nature of those exercises relative to the participant.

To the authors’ knowledge, this is the first study that compares the PAP effects of an EOL squat with TW squat exercise matching the concentric peak power. Therefore, a comparison between the current study and the literature is not possible. EOL and TW reported no meaningful differences in concentric peak power production, while EOL reported a *very strong* difference in eccentric peak power compared to TW. These results support the validity of the protocol used to match EOL and TW concentric intensities and underline also the EOL induced by flywheel exercise compared to TW. This greater eccentric load is a *peculiaritas* of flywheel exercises, since during the positive (extension) phase of a squat, the subject executes a high velocity movement (generally maximal) while during the negative (flexion) phase of the squat, the subject has to break the load accumulated during the previous phase.[1] Therefore, the principal advantage of EOL is related to an enchained mechanical load that is not possible using TW exercises. Authors supposed that a high eccentric load may have better stimulated higher order motor units (which require the utilization of high load), which may have guaranteed a positive transfer in motor unit recruitment, force, and power production during the following tasks (*e*.*g*. SLJ and CMJ) [7,32]. Additionally, acute performance improvements in sport-specific tasks may be associated with increased motor unit recruitment, rate coding, and neuromuscular inhibition [7,33]. Despite this strong theoretical rationale, this study found *moderate* effect in favor of H_0_ for PAP responses between the two pre-load exercises. Therefore, EOL and TW exercises, when matched for concentric peak power, reported equivalent PAP responses on SLJ, CMJ, and 5 m sprint performance. It is noteworthy that there is *moderate* statistical evidence in favor of similarity between the two methods (evidence in favor of H_0_). The results reported in the current study should be considered innovative since no previous studies have compared the PAP time windows following EOL and TW exercises. Furthermore, they may help strength and conditioning coaches to augment PAP strategies for athletes. Practitioners need to individualize recovery time and PAP onset obtained by EOL and TW exercises in order to enhance benefits from such strategies in competitions and complex training interventions [34]. Future studies on this argument are needed to confirm or contradict the findings of this study.

Existing literature reports that PAP effect magnitudes are related to the pre-load modality adopted. For instance, plyometric exercises seems to be more effective than both moderate and high intensity TW exercises, while maximal isometric contractions do not seem beneficial [8]. However, many factors may affect PAP such as the subject’s resistance training background (experienced vs inexperienced), as well as fitness level, where stronger individuals generally exhibit a larger PAP effect than weaker [8]. Moreover, PAP time window and magnitude may be related to the subjects’ muscle properties such as percentage of fast fibers [4,35]. Those factors should be further studied to understand the possible PAP differences between EOL and TW. Furthermore, the magnitude of the PAP effect could be different if an experienced (in strength training) cohort was enrolled. Such speculation may be supported by Dello Iacono et al. [31] that showed a PAP response (in acceleration tasks) following both moderate and intensive barbell hip trust exercises but that the effects differed according to the subject’s strength level. Authors may speculate that subjects’ device-specific resistance training background should be considered when selecting an exercise modality. For example, it is not known whether previous TW experience can be easily transferred to EOL exercise PAP response.

The current study is not without limitation. Firstly, this research has the assumption that PAP is the main explanation for the observed findings of improved performance but there is no explicit measurement of muscular activity and therefore direct evidence that PAP is the only mechanism underpinning such changes. Such limitation should be taken into consideration since the current research did not use a control group, but this design was utilized to reduce intrasession fatigue [7], furthermore because a possible placebo effect associated with the subjects’ knowledge of PAP could explain some changes. Secondly, this study compared EOL and TW exercise matching the intensity using the peak concentric power output. However, during the eccentric phase of EOL exercise the peak power was meaningfully greater than the eccentric peak power of TW exercise. Therefore, the total power (concentric and eccentric) generated by subjects was greater during the EOL than the TW squat exercise. Furthermore, practitioners need to consider access to EOL equipment in their daily practice, which could be less common than TW equipment. Lastly, this study enrolled a sample of amateur male athletes, therefore wider generalization cannot be inferred to other samples with different characteristics such as female athletes and professional athletes who may exhibit different PAP time window and magnitude responses [8,36,37].

## Conclusions

The present study suggests that both EOL and TW squat exercises acutely increase SLJ distance and CMJ height but not 5 m sprint performance in male amateur athletes. The onset of the PAP time window was found at 3 min following the protocol and the improvements in sport-specific tasks persisted at 7 min. This study did not find differences between EOL and TW exercises in PAP amplitude. Therefore, both exercise methodologies can be used to acutely stimulate PAP in a similar way before competitions and training sessions. Further research is needed to better clarify the similarities or differences in PAP time window and magnitude between EOL and TW squat exercise.

### Practical applications

Practitioners may use either EOL or TW squat exercises to stimulate a PAP response in athletes. Such acute potentiation has a positive effect on horizontal and vertical jumping performance, however, both protocols seem not to be efficient in improving sprinting acceleration performance. Future studies should explore this topic before drawing final conclusions, as well as clarifying the differences between the protocols. To optimize the PAP effect using EOL and TW pre-load methodologies (3 x 6 repetitions, with concentric peak power outputs of 1097 W and 1030 W, respectively), it is necessary to wait for 3 minutes following pre-load before initiating sport-specific movements; such PAP effects remain at least 7 min after completion of either pre-load strategy. Therefore, practitioners should consider such PAP time windows in sport-specific tasks before competitions or during training sessions (*e*.*g*. complex training). Furthermore, authors suggest individualizing the PAP protocol on the basis of athletes’ training experience, strength level, and morphological characteristics. This may help to optimize PAP as well as minimize acute fatigue and soreness. Authors suggest consideration of pre-load exercise (EOL or TW) on the basis of athletes’ previous strength training experience with such protocols.
